# Exhausted and Senescent T Cells at the Maternal-Fetal Interface in Preterm and Term Labor

**DOI:** 10.1155/2019/3128010

**Published:** 2019-05-23

**Authors:** Rebecca Slutsky, Roberto Romero, Yi Xu, Jose Galaz, Derek Miller, Bogdan Done, Adi L. Tarca, Sabrina Gregor, Sonia S. Hassan, Yaozhu Leng, Nardhy Gomez-Lopez

**Affiliations:** ^1^Perinatology Research Branch, Division of Obstetrics and Maternal-Fetal Medicine, Division of Intramural Research, Eunice Kennedy Shriver National Institute of Child Health and Human Development, National Institutes of Health, U.S. Department of Health and Human Services, Bethesda, Maryland 20892 and Detroit, Michigan 48201, USA; ^2^Department of Obstetrics and Gynecology, University of Michigan, Ann Arbor, Michigan 48109, USA; ^3^Department of Epidemiology and Biostatistics, Michigan State University, East Lansing, Michigan 48824, USA; ^4^Center for Molecular Medicine and Genetics, Wayne State University, Detroit, Michigan 48201, USA; ^5^Department of Obstetrics and Gynecology, Wayne State University School of Medicine, Detroit, Michigan 48201, USA; ^6^Department of Obstetrics and Gynecology, Faculty of Medicine, Pontificia Universidad Católica de Chile, Santiago 8330024, Chile; ^7^Department of Physiology, Wayne State University School of Medicine, Detroit, Michigan 48201, USA; ^8^Department of Immunology, Microbiology and Biochemistry, Wayne State University School of Medicine, Detroit, Michigan 48201, USA

## Abstract

Successful pregnancy requires a tightly-regulated equilibrium of immune cell interactions at the maternal-fetal interface (i.e., the decidual tissues), which plays a central role in the inflammatory process of labor. Most of the innate immune cells in this compartment have been well characterized; however, adaptive immune cells are still under investigation. Herein, we performed immunophenotyping of the decidua basalis and decidua parietalis to determine whether exhausted and senescent T cells are present at the maternal-fetal interface and whether the presence of pathological (i.e., preterm) or physiological (i.e., term) labor and/or placental inflammation alter such adaptive immune cells. In addition, decidual exhausted T cells were sorted to test their functional status. We found that (1) exhausted and senescent T cells were present at the maternal-fetal interface and predominantly expressed an effector memory phenotype, (2) exhausted CD4^+^ T cells increased in the decidua parietalis as gestational age progressed, (3) exhausted CD4^+^ and CD8^+^ T cells decreased in the decidua basalis of women who underwent labor at term compared to those without labor, (4) exhausted CD4^+^ T cells declined with the presence of placental inflammation in the decidua basalis of women with preterm labor, (5) exhausted CD8^+^ T cells decreased with the presence of placental inflammation in the decidua basalis of women who underwent labor at term, (6) both senescent CD4^+^ and CD8^+^ T cells declined with the presence of placental inflammation in the decidua basalis of women who underwent preterm labor, and (7) decidual exhausted T cells produced IFN*γ* and TNF*α* upon *in vitro* stimulation. Collectively, these findings indicate that exhausted and senescent T cells are present at the human maternal-fetal interface and undergo alterations in a subset of women either with labor at term or preterm labor and placental inflammation. Importantly, decidual T cell function can be restored upon stimulation.

## 1. Introduction

Successful pregnancy requires that the mother and semiallogeneic fetus coexist, which involves systemic and local (i.e., maternal-fetal interface) immune interactions [1–9]. The maternal-fetal interface (i.e., the decidua) is formed after the endometrium undergoes morphological and functional changes (“decidualization”), allowing for invasion of fetal trophoblast and forming the area of contact between the endometrium and the placenta (decidua basalis) or chorioamniotic membranes (decidua parietalis) [10, 11]. The major immune cell types present at the maternal-fetal interface [7, 12] include components of the innate limb such as natural killer (NK) cells [13–17], macrophages [18–27], neutrophils [28, 29], and the recently described innate lymphoid cells [30–35]. The adaptive immune cells, T cells [36–50] and B cells [51–54], are also present at the maternal-fetal interface. A tightly-regulated equilibrium between these immune cells is required for pregnancy maintenance [6, 7], and a disruption of this balance may lead to pregnancy complications such as preterm labor and birth [55, 56], the leading cause of neonatal mortality and morbidity worldwide [57–59]. Specifically, we have recently shown that a pool of effector and activated decidual T cells leads to pathological inflammation resulting in spontaneous preterm labor and birth [60, 61]. However, whether decidual T cells undergo a process of exhaustion (exhausted T cells [62–69]) or senescence (senescent T cells [70–72]), which leads to a loss of function, is unknown. To date, there is no evidence of exhausted or senescent T cells at the human maternal-fetal interface.

T cell exhaustion results from continuous exposure to antigen and occurs as a progressive loss of function, characterized by increased coexpression of multiple inhibitory receptors (e.g., TIM-3, PD-1, CTLA-4, and LAG-3), changes in the expression of transcription factors, distinctive patterns of cytokine receptors, loss of effector cytokine secretion, and metabolic alterations [68, 69, 73]. A key hallmark of exhausted T cells is the lack of canonical memory T cell properties and maintenance [73]. In humans, T cell exhaustion was described during chronic viral infections [e.g., human immunodeficiency virus (HIV), hepatitis B virus (HBV), and hepatitis C virus (HCV)] as well as in cancer [68, 69, 73, 74]. T cell exhaustion has also been implicated in the mechanisms of allograft or transplant tolerance [75–77]. However, whether T cell exhaustion is implicated in pregnancy complications such as preterm labor and birth is unknown.

T cell exhaustion has been related to T cell senescence as both processes involve cell dysfunction [78]. However, it is now clear that these cell fates are distinct and regulated independently of each other [78]. Senescent T cells lose their proliferative capacity while maintaining effector functions (i.e., cytokine production and cytotoxicity) [78], whereas exhausted T cells have typically lost both proliferative capacity and the majority of their functions [65]. In addition, senescent T cells express high levels of CD57 and KLRG-1 [79, 80], while expression of these markers is low on exhausted T cells [65, 68]. Moreover, exhausted T cells have high expression of inhibitory receptors, whereas senescent cells do not [73]. Given that T cell exhaustion is being investigated herein, we also determined whether senescent T cells are present at the maternal-fetal interface and whether such cells are associated with preterm labor and birth.

**Figure 1:**
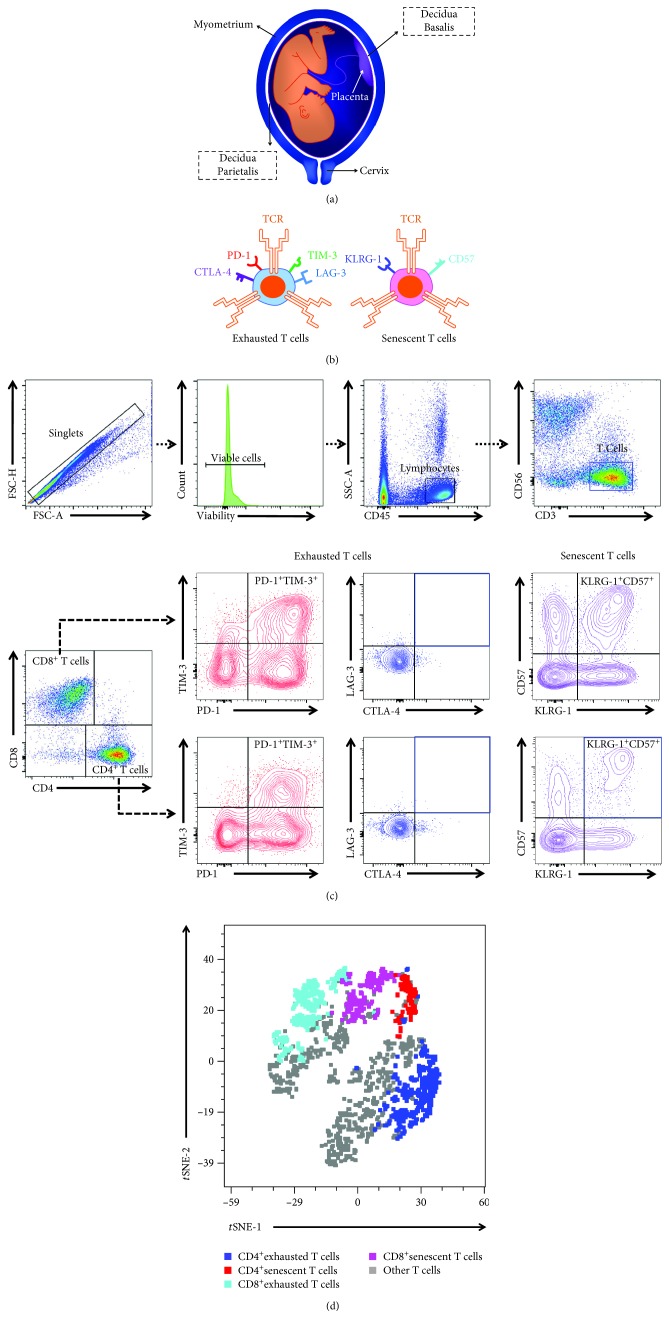
Immunophenotyping of exhausted and senescent T cells in the decidua basalis and decidua parietalis. (a) Representation of the spatial localization of the decidua basalis and decidua parietalis. (b) Schematic representation of select markers expressed by exhausted and senescent T cells. (c) Flow cytometry gating strategy used to identify exhausted and senescent T cells in the decidual tissues. T cells were gated as CD3^+^CD56^−^ cells within the viability and lymphocytic gates, followed by gating for the CD4^+^ and CD8^+^ subsets. Exhausted T cells were gated for expression of PD-1, TIM-3, CTLA-4, and LAG-3. Since expression of CTLA-4 and LAG-3 was low, exhausted T cells were defined as PD-1^+^TIM-3^+^ cells within the CD4^+^ or CD8^+^ gates. Senescent T cells were gated as KLRG-1^+^CD57^+^ cells within the CD4^+^ or CD8^+^ gates. (d) A representative *t*-distributed stochastic neighbor embedding (*t*-SNE) dot plot visualizing exhausted and senescent CD4^+^ and CD8^+^ T cells among decidual T cells. Blue—CD4^+^ exhausted T cells, red—CD4^+^ senescent T cells, turquoise—CD8^+^ exhausted T cells, pink—CD8^+^ senescent T cells, and grey—other T cells.

In the current study, we performed immunophenotyping of the maternal-fetal interface (i.e., the decidua basalis and decidua parietalis; Figure 1(a)) to determine whether exhausted and senescent T cells are present in preterm and term gestations. In addition, we investigated whether the presence of pathological (i.e., preterm) or physiological (i.e., term) labor and/or placental inflammation alter exhausted and senescent T cells at the maternal-fetal interface. Lastly, decidual exhausted T cells were sorted and their functionality was tested *in vitro*.

## 2. Materials and Methods

### 2.1. Human Subjects, Clinical Specimens, and Definitions

Human placental basal plate (decidua basalis) and chorioamniotic membrane (amnion, chorion, and decidua parietalis) samples were collected from patients within 30 min after delivery at Hutzel Women's Hospital in the Detroit Medical Center, Detroit, MI, USA, in partnership with Wayne State University School of Medicine and the Perinatology Research Branch, an intramural program of the *Eunice Kennedy Shriver* National Institute of Child Health and Human Development, National Institutes of Health, US Department of Health and Human Services (NICHD/NIH/DHHS), Detroit, MI, USA. The collection and utilization of biological materials for research purposes were approved by the Institutional Review Boards of Wayne State University and NICHD. All participating women provided written informed consent prior to sample collection. The study groups included women who delivered at term with (TIL) or without (TNL) labor and women who delivered preterm with (PTL) or without (PTNL) labor. Preterm birth was defined as delivery before 37 weeks of gestation, and term birth was defined as delivery after 37 weeks of gestation. Labor was defined by the presence of regular uterine contractions at a frequency of at least 2 contractions every 10 min with cervical changes resulting in delivery. The TIL and PTL study groups were subdivided based on the presence of placental inflammation (PI) in the chorioamniotic membranes (see Placental Histopathological Examination for diagnostic criteria). The clinical and demographic characteristics of the study population are shown in Tables 1 and 2.

### 2.2. Placental Histopathological Examination

Placentas were examined histologically by a perinatal pathologist blinded to clinical diagnoses and obstetrical outcomes according to standardized Perinatology Research Branch protocols [81]. Briefly, three to nine sections of the placenta were examined, and at least one full-thickness section was taken from the center of the placenta; others were taken randomly from the placental disc. Inflammatory lesions of the placenta were diagnosed according to established criteria [82–84]. Placental inflammation was defined by the infiltration of neutrophils into the chorion and amnion [83].

### 2.3. Isolation of Decidual Leukocytes

Decidual leukocytes were isolated from the decidua basalis and decidua parietalis as previously described [85]. Briefly, the decidua basalis was collected from the basal plate of the placenta and the decidua parietalis was separated from the chorioamniotic membranes (Figure 1(a)). The decidual tissues were homogenized using a gentleMACS Dissociator (Miltenyi Biotec, San Diego, CA, USA) in StemPro Accutase Cell Dissociation Reagent (Life Technologies, Grand Island, NY, USA). Homogenized tissues were incubated for 45 min at 37°C with gentle agitation. After incubation, tissues were washed in sterile 1X phosphate-buffered saline (PBS) (Life Technologies) and filtered through a 100 *μ*m cell strainer (Falcon, Corning Life Sciences Inc., Durham, NC, USA). The resulting cell suspension was centrifuged at 300 x g for 10 min at 4°C. Decidual leukocytes were then separated using a density gradient (Ficoll-Paque Plus; GE Healthcare Biosciences, Uppsala, Sweden), following the manufacturer's instructions. The cells collected from the mononuclear layer of the density gradient were washed with 1X PBS and immediately used for immunophenotyping.

### 2.4. Immunophenotyping of Decidual T Cells

Isolated decidual mononuclear cells were incubated with BD Fixable Viability Stain 575V (Cat#565694; BD Biosciences, San Jose, CA, USA) for 30 min at 4°C, then washed with 1X PBS. Next, the cells were resuspended in 50 *μ*L of stain buffer (BD Biosciences) and incubated with fluorochrome-conjugated anti-human monoclonal antibodies (Supplementary Table 1) for 30 min at 4°C in the dark. After extracellular staining, the cells were washed with 1X PBS to remove excess antibody, resuspended in 0.5 mL of stain buffer, and acquired using the BD LSRFortessa Flow Cytometer (BD Biosciences) and BD FACSDiva 6.0 software (BD Biosciences). The analysis and figures were performed using FlowJo software version 10 (FlowJo, LLC, Ashland, OR, USA). The cell surface markers used to identify exhausted and senescent T cells were selected based on a literature review (Supplementary Table 2). The effector memory status of exhausted and senescent T cells was determined by the expression of CD45RA and CCR7.

### 2.5. Cytokine Production by Decidual Exhausted T Cells

Decidual mononuclear cells were isolated as described above and incubated with BD Fixable Viability Stain 510 (Cat#564406; BD Biosciences) for 30 min at 4°C, then washed with 1X PBS. The cells were then resuspended in 50 *μ*L of stain buffer and incubated with fluorochrome-conjugatedanti-human monoclonal antibodies (Supplementary Table 1) for 30 min at 4°C in the dark. After extracellular staining, the cells were washed with 1X PBS to remove excess antibody, resuspended in 0.5 mL of presort buffer (Cat#563503; BD Biosciences), and exhausted CD4^+^ (CD45^+^CD3^+^CD4^+^Tim-3^+^PD-1^+^ cells) and CD8^+^ (CD45^+^CD3^+^CD8^+^Tim-3^+^PD-1^+^ cells) T cells were sorted using the BD FACSMelody cell sorter (BD Biosciences) and BD FACSChorus version 1.3 software (BD Biosciences). For the determination of T cell function, sorted exhausted T cells were stimulated for 4 h with 2 *μ*L/mL of Cell Stimulation Cocktail [phorbol 12-myristate 13-acetate (PMA), ionomycin, brefeldin A, and monensin (Cat#00-4975; Life Technologies)]. Stimulated exhausted T cells were then collected, fixed, and permeabilized using the BD Cytofix/Cytoperm Fixation and Permeabilization Solution (BD Biosciences) and incubated with specific monoclonal antibodies against IFN*γ* and TNF*α* (Supplementary Table 1). Nonstimulated sorted exhausted T cells were used as controls. Stained exhausted T cells were acquired using the BD LSRFortessa Flow Cytometer and BD FACSDiva 6.0 software. The analysis and figures were performed using FlowJo version 10 software (FlowJo).

### 2.6. Statistical Analysis

Data were analyzed using IBM SPSS version 19.0 (IBM Corporation; Armonk, NY, USA). For patient demographics, the Fisher's exact test was used to compare proportions among groups and the Kruskal-Wallis test was used to compare continuous variables among groups. Experimental data were compared between study groups using the Mann-Whitney *U*-test. Two-tailed (*p* values without an asterisk) and one-tailed (*p* values with an asterisk) *p* values were reported. The *t*-distributed stochastic neighbor embedding (*t*-SNE) plot was generated using FlowJo version 10 software. The association between exhausted and senescent T cells and gestational age was assessed using a Spearman's correlation test. *p* values were adjusted across the T cell subsets using the false discovery rate method [86]. Nonparametric local weighted regression (LOESS) [87] was used to estimate the average percentage of each T cell subset as a function of gestational age. The R statistical package was used for analysis [88]. A *p* value ≤ 0.05 was considered statistically significant.

## 3. Results

### 3.1. Exhausted and Senescent T Cells Are Present at the Maternal-Fetal Interface

Figure 1(a) shows the spatial localization of the decidua basalis and decidua parietalis. The markers for the identification of exhausted and senescent T cells are shown in Figure 1(b). The gating strategy used to identify exhausted and senescent CD4^+^ and CD8^+^ T cells in the decidua basalis and decidua parietalis is shown in Figure 1(c). In the decidual tissues, exhausted CD4^+^ and CD8^+^ T cells expressed PD-1 and TIM-3, but lacked expression of LAG-3 and CTLA-4. We considered exhausted T cells as those expressing both PD-1 and TIM-3 (Figure 1(c)). In the decidual tissues, we considered senescent CD4^+^ and CD8^+^ T cells as those expressing both KLRG-1 and CD57 (Figure 1(c)). A *t*-SNE plot representing the abundance of exhausted and senescent CD4^+^ and CD8^+^ T cells among decidual T cells is shown in Figure 1(d).

**Figure 2:**
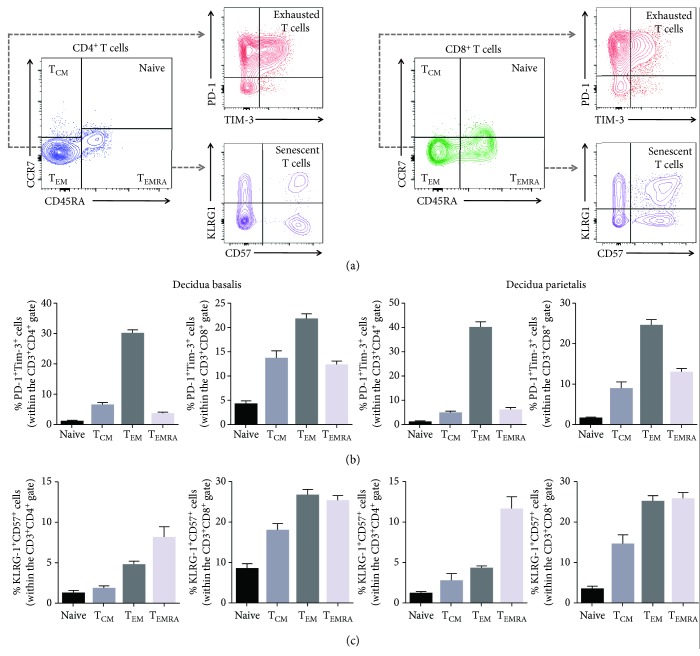
Proportions of exhausted and senescent CD4^+^ and CD8^+^ decidual T cells within the effector memory subsets. (a) Flow cytometry gating strategy used to identify exhausted and senescent decidual CD4^+^ and CD8^+^ T cells within the naïve, central memory (T_CM_), effector memory (T_EM_), and terminally differentiated effector memory (T_EMRA_) subsets. (b) Proportions of exhausted CD4^+^ and CD8^+^ T cells within the naïve, T_CM_, T_EM_, and T_EMRA_ subsets in the decidua basalis and decidua parietalis. (c) Proportions of senescent CD4^+^ and CD8^+^ T cells within the naïve, T_CM_, T_EM_, and T_EMRA_ subsets in the decidua basalis and decidua parietalis. *N* = 55. Data are shown as the means with a standard error of the mean.

The majority of exhausted CD4^+^ and CD8^+^ T cells belong to the effector memory T cell subset (T_EM_) in the decidua basalis and decidua parietalis (Figures 2(a) and 2(b)). Yet, some of the exhausted CD8^+^ T cells were also found in the central memory (T_CM_) and terminally differentiated effector memory (T_EMRA_) subsets (Figure 2(b)). Most of the senescent CD4^+^ T cells belonged to the T_EMRA_ subset, whereas senescent CD8^+^ T cells were found in both the T_EM_ and T_EMRA_ subsets in the decidua basalis and decidua parietalis (Figures 2(a) and 2(c)).

Together, these findings indicate that exhausted and senescent T cells are found at the maternal-fetal interface, where most of them express an effector memory phenotype.

### 3.2. Exhausted CD4^+^ and CD8^+^ T Cells Increase in the Decidua Parietalis as Gestational Age Progresses

**Figure 3:**
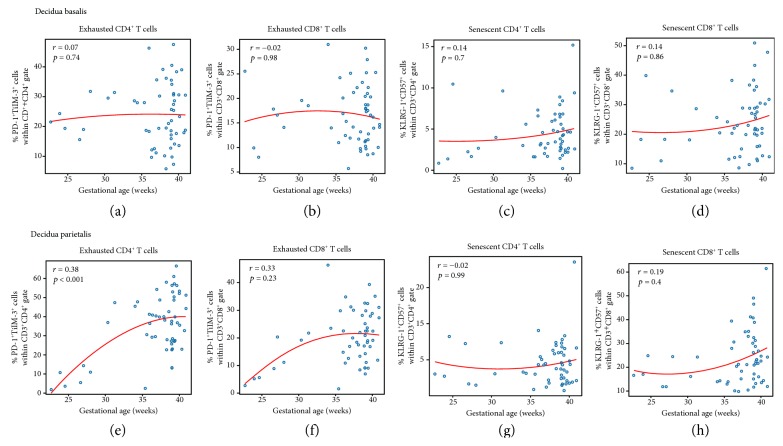
Correlations between exhausted or senescent CD4^+^ and CD8^+^ decidual T cells and gestational age. The correlations between gestational age and the proportions of exhausted or senescent CD4^+^ and CD8^+^ T cells in the decidua basalis (a–d) and decidua parietalis (e–h). The red line represents locally weighted scatter plot smoothing (LOESS) estimating the average cell percentages as a function of gestational age (weeks). The correlations were assessed using a Spearman's correlation test. Correlation coefficients and *p* values are shown for each plot.

Next, we determined whether the abundance of exhausted or senescent T cells changes as gestational age advances, given that the T cell repertoire undergoes alterations throughout gestation [12]. The Spearman correlations between the proportions of exhausted or senescent CD4^+^ and CD8^+^ T cells and gestational age are shown in Figure 3. In the decidua basalis, no significant correlations were observed between exhausted or senescent CD4^+^ and CD8^+^ T cells and gestational age (Figures 3(a)–3(d)). In the decidua parietalis, exhausted CD4^+^ T cells significantly increased from preterm to term gestation (*p* < 0.001; Figure 3(e)). The same positive correlation was observed for exhausted CD8^+^ T cells, yet this did not reach a statistical significance (Figure 3(f)). In the decidua parietalis, senescent CD4^+^ and CD8^+^ T cells did not vary as gestational age progressed (Figures 3(g) and 3(h)). These data show that the abundance of exhausted CD4^+^ and CD8^+^ T cells in the decidua parietalis increases as gestational age progresses.

### 3.3. Exhausted CD4^+^ and CD8^+^ T Cells Decrease in the Decidua Basalis of Women with Labor at Term

**Figure 4:**
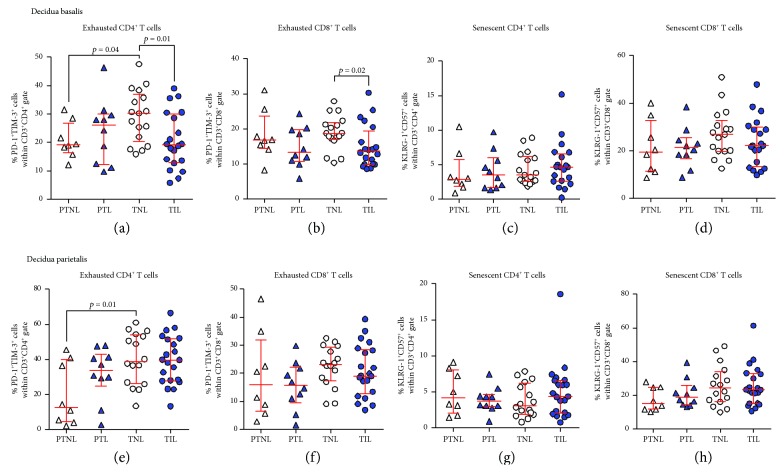
Proportions of exhausted and senescent CD4^+^ and CD8^+^ T cells in the decidua basalis and decidua parietalis. The proportions of exhausted and senescent CD4^+^ and CD8^+^ T cells in the decidua basalis (a–d) and decidua parietalis (e–h) from women who delivered preterm with labor (PTL) or without labor (PTNL) and women who delivered at term with labor (TIL) or without labor (TNL). *N* = 8–21 per group. Red midlines and whiskers indicate medians and interquartile ranges, respectively.

Our previous studies have suggested that T cells participate in the physiological [45, 46, 89, 90] and pathological [56, 60, 61, 91, 92] processes of labor (i.e., labor at term and preterm labor). Therefore, we investigated whether exhausted and senescent T cells were altered with the presence of labor at term or preterm labor. In the decidua basalis, exhausted CD4^+^ and CD8^+^ T cells were reduced in women who underwent labor at term compared to those who delivered at term without labor (Figures 4(a) and 4(b)). However, this reduction was not observed when comparing the preterm labor and preterm without labor groups (Figures 4(a) and 4(b)). In the decidua basalis, senescent CD4^+^ and CD8^+^ T cells did not vary between the labor and nonlabor groups (Figures 4(c) and 4(d)). In the decidua parietalis, exhausted and senescent CD4^+^ and CD8^+^ T cells did not vary between the labor and nonlabor groups at term and preterm gestations (Figures 4(e)–4(h)). Consistent with our previous results, in the absence of labor, exhausted CD4^+^ T cells were more abundant in the term than in the preterm groups (Figures 4(a) and 4(e)). Similar differences in exhausted and senescent T cells between the study groups were observed when such cells were gated within the effector memory subsets (Supplementary Figure 1A–1L). Taken together, these data indicate that the physiological process of labor at term, but not the pathological process of preterm labor, is accompanied by a decline in exhausted CD4^+^ and CD8^+^ T cells at the maternal-fetal interface.

### 3.4. The Impact of Placental Inflammation on Exhausted and Senescent CD4^+^ T Cells in the Decidual Tissues

Pathological inflammation is associated with an imbalance between immune cells at the maternal-fetal interface [56]. Thus, we next evaluated whether inflammation in the placenta of women who underwent preterm labor or labor at term impacted the abundance of exhausted or senescent T cells in the decidual tissues.

**Figure 5:**
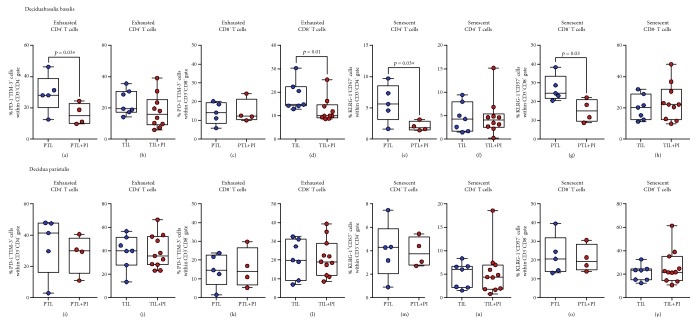
Proportions of exhausted and senescent CD4^+^ and CD8^+^ T cells in the decidua basalis and decidua parietalis with placental inflammation. The proportions of exhausted and senescent CD4^+^ and CD8^+^ T cells in the decidua basalis (a–h) and decidua parietalis (i–p) of women who underwent preterm labor with (PTL+PI) or without (PTL) placental inflammation or labor at term with (TIL+PI) or without (TIL) placental inflammation. *N* = 4–11. Midlines—medians, boxes—interquartile ranges, and whiskers—minimum and maximum ranges. PI: placental inflammation.

Exhausted CD4^+^ T cells, but not exhausted CD8^+^ T cells, were reduced in the decidua basalis of women who underwent preterm labor with placental inflammation compared to those without this condition (Figures 5(a) and 5(c)). In contrast, exhausted CD8^+^ T cells, but not exhausted CD4^+^ T cells, were decreased in the decidua basalis of women who underwent labor at term with placental inflammation compared to those without inflammation (Figures 5(b) and 5(d)). Both senescent CD4^+^ and CD8^+^ T cells were reduced in the decidua basalis of women who underwent preterm labor with placental inflammation compared to those without this condition (Figures 5(e) and 5(g)). However, senescent CD4^+^ and CD8^+^ T cells in the decidua basalis did not vary between term labor women with and without placental inflammation (Figures 5(f) and 5(h)). Placental inflammation did not alter the abundance of exhausted or senescent CD4^+^ and CD8^+^ T cells in the decidua parietalis (Figures 5(i)–5(p)). These findings show that placental inflammation can selectively impact the abundance of exhausted and senescent T cells in the decidua basalis of women who underwent preterm labor or labor at term.

### 3.5. Decidual Exhausted T Cells Are Functional upon *In Vitro* Stimulation

**Figure 6:**
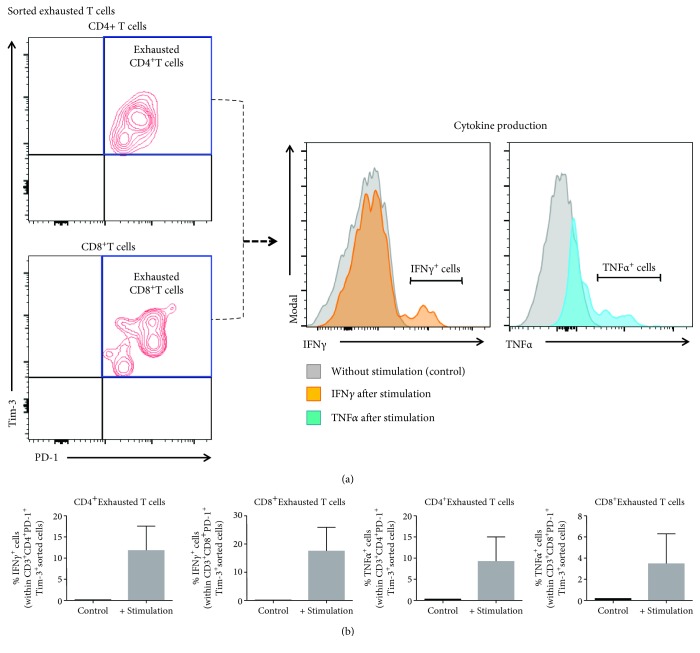
Determination of exhausted T cell functionality upon *in vitro* stimulation. (a) Gating strategy used to determine the purity of sorted CD4^+^ and CD8^+^ exhausted decidual T cells. Histograms show the production of IFN*γ* (orange histogram) and TNF*α* (blue histogram) by sorted exhausted T cells after *in vitro* stimulation. Grey histograms indicate nonstimulated controls. (b) Proportions of sorted exhausted CD4^+^ and CD8^+^ decidual T cells expressing IFN*γ* or TNF*α* after *in vitro* stimulation compared to nonstimulated controls. *N* = 3. Data are shown as the means with a standard error of the mean.

Exhausted T cells lose their effector functions, whereas senescent T cells do not [78]. Therefore, we sorted exhausted T cells from the decidual tissues and tested their functionality upon *in vitro* stimulation. The purity of sorted exhausted T cells is shown in Figure 6(a). Functionality was tested by the production of IFN*γ* and TNF*α* (Figure 6(a)). Consistent with our *in vivo* data (e.g., reduction of exhausted T cells in placental inflammation), exhausted T cells produced inflammatory cytokines upon *in vitro* stimulation, suggesting the restoration of an effector phenotype (Figure 6(b)). These data imply that exhausted T cells restore their functional-effector phenotype during inflammatory conditions at the maternal-fetal interface.

## 4. Discussion

### 4.1. Principal Findings

The principal findings of this study are as follows: (1) exhausted and senescent T cells were present at the human maternal-fetal interface and predominantly expressed an effector memory phenotype; (2) exhausted CD4^+^ T cells increased in the decidua parietalis as gestational age progressed; (3) exhausted CD4^+^ and CD8^+^ T cells decreased in the decidua basalis of women who underwent labor at term compared to those without labor; (4) exhausted CD4^+^ T cells declined with the presence of placental inflammation in the decidua basalis of women with preterm labor; (5) exhausted CD8^+^ T cells decreased with the presence of placental inflammation in the decidua basalis of women who underwent labor at term; (6) both senescent CD4^+^ and CD8^+^ T cells declined with the presence of placental inflammation in the decidua basalis of women who underwent preterm labor; and (7) decidual exhausted T cells produced IFN*γ* and TNF*α* upon *in vitro* stimulation. Together, these findings indicate that exhausted and senescent T cells are present at the maternal-fetal interface and undergo alterations in a subset of women either with labor at term or preterm labor and placental inflammation, yet can restore their functionality upon stimulation.

### 4.2. Exhausted T Cells at the Maternal-Fetal Interface in Term and Preterm Labor

Herein, for the first time, we identified exhausted CD4^+^ and CD8^+^ T cells at the human maternal-fetal interface. Such cells display an effector memory phenotype, consistent with that of other tissue-resident exhausted T cells [93, 94]. Recent studies have identified decidual T cells expressing PD-1 and TIM-3 during the first trimester [95, 96] and in term pregnancy [50, 97]. However, the abovementioned studies did not identify such cells as exhausted T cells. It is thought that T cells expressing PD-1 and TIM-3 participate in the mechanisms leading to immune tolerance [76, 77, 98–100]; therefore, such molecules have been implicated in the pathophysiology of pregnancy loss [101–105]. The fact that decidual exhausted T cells expressing PD-1 and TIM-3 are more abundant in term than in preterm gestations suggests that T cell dysfunction represents a regulatory mechanism to prevent exacerbated cellular responses toward the end of pregnancy.

We and others have found that the lack of functionality by decidual T cells can be restored *in vitro* [50], suggesting that the inflammatory milieu that accompanies the physiological process of labor at term [106–113] reinvigorates T cell responses (i.e., reversal of T cell exhaustion [114]) at the maternal-fetal interface. This concept could explain why women who underwent labor at term had reduced proportions of exhausted T cells compared to those who delivered at term without labor.

In the current study, no differences in exhausted T cells were found in the decidual tissues of women who underwent preterm labor compared to those who delivered preterm without labor. This finding supports the hypothesis that the pathological process of preterm labor is distinct from the physiological process of labor at term [115–119] and that, in most cases, occurs in the absence of a reduction in T cell exhaustion. However, acute placental inflammation (the only causal link to spontaneous preterm labor [120–127] and present in a subset of women who deliver preterm [128–131]) decreased the abundance of exhausted T cells at the maternal-fetal interface, suggesting that T cell exhaustion is reduced solely in some cases of preterm labor associated with exacerbated placental inflammation. The mechanisms whereby placental inflammation can reduce T cell exhaustion at the maternal-fetal interface may involve cytokines, given that such inflammatory mediators can reverse T cell dysfunction [73, 132–135]. Therefore, we surmise that placental inflammation boosts effector T cell function by dampening T cell exhaustion at the maternal-fetal interface in a subset of women who undergo preterm labor.

A central question that arises from this study is whether T cell exhaustion at the maternal-fetal interface can be augmented in order to ameliorate effector T cell responses that lead to pathological inflammation and preterm labor and birth. T cell exhaustion has been manipulated by targeting the TCR and inhibitory receptors (e.g., PD-1, TIM-3, CTLA-4, and LAG-3) as well as by treatment with soluble mediators (e.g., anti-inflammatory cytokines such as IL-10 and TGF*β*) and suppressive cells [67, 68, 135–141]. Further research is required to investigate which of the abovementioned strategies could be safely utilized during pregnancy.

### 4.3. Senescent T Cells at the Maternal-Fetal Interface in Preterm Labor

To our knowledge, we are the first to identify senescent T cells at the human maternal-fetal interface. Decidual senescent T cells express an effector memory phenotype consistent with that displayed by these cells in other tissues [80]. Unlike exhausted T cells, senescent T cells can release proinflammatory mediators such as IFN*γ*, TNF*α*, granzyme B, and perforin [80, 142, 143]. We and others have shown that T cells can release such inflammatory mediators at the maternal-fetal interface [50, 61], suggesting that senescent T cells may contribute to the inflammatory milieu in this microenvironment.

We also found that senescent T cells were reduced in women who underwent preterm labor associated with placental inflammation. This finding is in line with the hypothesis that cellular senescence is implicated in the mechanisms of disease for preterm labor and birth [55, 144, 145]. The mechanisms whereby placental inflammation reduces senescent T cells at the maternal-fetal interface of women with preterm labor may involve the p53 pathway, mitogen-activated protein kinase p38 (MAPKp38), and the cyclin-dependent kinase inhibitors p16 and p21 [78], all of which are implicated in the process of parturition [144–150]. Given that T cells can undergo reversible senescence [71, 78, 143, 151–153], additional research is required to investigate the mechanisms implicated in such a process at the maternal-fetal interface.

It is worth mentioning that the effect of gestational age was observed in the decidua parietalis, whereas the impact of the process of labor and placental inflammation was mainly observed in the decidua basalis. This finding exemplifies the complexity of the maternal-fetal interface and highlights the importance of considering both the maternal (i.e., decidua parietalis is in contact with the endometrium) and fetal (i.e., decidua basalis is attached to the placenta) sides when studying maternal-fetal interactions.

## 5. Conclusion

In the current study, exhausted and senescent effector memory T cells were identified at the human maternal-fetal interface, where they are more abundant as term approaches. To our knowledge, this is the first time that exhausted T cells have been identified at the human maternal-fetal interface. While the physiological process of labor at term was associated with a decline in exhausted T cells, the pathological process of preterm labor with placental inflammation was linked to a reduction in both exhausted and senescent T cells. Moreover, we show that exhausted T cells restore their functionality upon *in vitro* stimulation. Collectively, these data suggest that exhausted and senescent T cells are physiological components of the maternal-fetal interface and that such cells play a role in homeostasis and disease during pregnancy.

## Figures and Tables

**Table 1:** Clinical and demographic characteristics of the patient population used to perform immunophenotyping of exhausted and senescent T cells in the decidua basalis.

	Term without labor (*n* = 17)	Term with labor (*n* = 20)	Preterm without labor (*n* = 8)	Preterm with labor (*n* = 10)	*p* value
Maternal age (years; median (IQR))^a^	26 (25-32)	23.5 (21-26.3)	28 (25.3-30.8)	22.5 (21-31.8)	0.04
Body mass index (kg/m^2^; median (IQR))^a^	30.1 (26-36.1)^c^	24.7 (23.1-33.5)	32.9 (22.7-42.9)	25.7 (20.5-27.4)^c^	0.3
Primiparity^b^	11.8% (2/17)	35% (7/20)	12.5% (1/8)	20% (2/10)	0.3
Race^b^					0.1
African-American	68.8% (11/16)^c^	90% (18/20)	75% (6/8)	90% (9/10)	
Caucasian	18.8% (3/16)^c^	0% (0/20)	12.5%(1/8)	0% (0/10)	
Asian	12.5% (2/16)^c^	0% (0/20)	0% (0/8)	0% (0/10)	
Other	0% (0/16)^c^	10% (2/20)	12.5% (1/8)	10% (1/10)	
Gestational age at delivery (weeks; median (IQR))^a^	39.1 (39-39.3)	39.2 (38.5-40)	27.6 (26.1-34.5)	35.5 (32.1-36.2)	<0.001
Birthweight (g)^a^	2960 (2775-3285)	3195 (2925-3693.8)	728.5 (595-2078.8)	2305 (1656.3-2446.3)	<0.001
Cesarean section^b^	100% (17/17)	35% (7/20)	100% (8/8)	40% (4/10)	<0.001

Data are given as the median (interquartile range) and percentage (*n*/*N*). ^a^Kruskal-Wallis test. ^b^Fisher's exact test. ^c^One missing data.

**Table 2:** Clinical and demographic characteristics of the patient population used to perform immunophenotyping of exhausted and senescent T cells in the decidua parietalis.

	Term without labor (*n* = 16)	Term with labor (*n* = 21)	Preterm without labor (*n* = 8)	Preterm with labor (*n* = 10)	*p* value
Maternal age (years; median (IQR))^a^	27 (25-32.3)	24 (21-26)	28 (25.3-30.8)	22.5 (21-31.8)	0.05
Body mass index (kg/m^2^; median (IQR))^a^	30.1 (27-36.9)^c^	23.5 (23-32.8)	32.9 (22.7-42.9)	25.7 (20.5-27.4)^c^	0.2
Primiparity^b^	12.5% (2/16)	38.1% (8/21)	12.5% (1/8)	20% (2/10)	0.3
Race^b^					0.09
African-American	66.7% (10/15)^c^	90.5% (19/21)	75% (6/8)	90% (9/10)	
Caucasian	20% (3/15)^c^	0% (0/21)	12.5% (1/8)	0% (0/10)	
Asian	13.3% (2/15)^c^	0% (0/21)	0% (0/8)	0% (0/10)	
Other	0% (0/15)^c^	9.5% (2/21)	12.5% (1/8)	10% (1/10)	
Gestational age at delivery (weeks; median (IQR))^a^	39.1 (39-39.3)	39.3 (38.6-40)	27.6 (26.1-34.5)	35.5 (32.1-36.2)	<0.001
Birthweight (g)^a^	2972.5 (2763.8-3290)	3295 (2935-3675)	728.5 (595-2078.8)	2305 (1656.3-2446.3)	<0.001
Cesarean section^b^	100% (16/16)	33.3% (7/21)	100% (8/8)	40% (4/10)	<0.001

Data are given as the median (interquartile range) and percentage (*n*/*N*). ^a^Kruskal-Wallis test. ^b^Fisher's exact test. ^c^One missing data.

## Data Availability

The data used to support the findings of this study are available from the corresponding author upon request.

## Supplementary Materials

Supplementary Materials Supplementary Table 1: antibodies used for immunophenotyping and cell sorting. Supplementary Table 2: literature review performed to select markers for identifying exhausted and senescent T cells. Supplementary Figure 1: exhausted and senescent CD4^+^ and CD8^+^ T cells display an effector memory phenotype in the decidua basalis and decidua parietalis. The proportions of exhausted and senescent CD4^+^ and CD8^+^ T cells within the effector memory (TEM) (A-H) or terminally differentiated effector memory (TEMRA) (I-L) T cell subsets in the decidua basalis and decidua parietalis from women who delivered preterm with labor (PTL) or without labor (PTNL) and women who delivered at term with labor (TIL) or without labor (TNL). *N* = 8–21 per group. Red midlines and whiskers indicate medians and interquartile ranges, respectively.

## References

[1] Medawar P. B. (1953). Some immunological and endocrinological problems raised by the evolution of viviparity in vertebrates. *Symposia of the Society for Experimental Biology*.

[2] Finn R., St Hill C. A., Davis J. C., Hipkin L. J., Harvey M. (1977). Feto-maternal bidirectional mixed lymphocyte reaction and survival of fetal allograft. *The Lancet*.

[3] Chaouat G., Kolb J. P., Wegmann T. G. (1983). The murine placenta as an immunological barrier between the mother and the fetus. *Immunological Reviews*.

[4] Hunziker R. D., Wegmann T. G. (1986). Placental immunoregulation. *Critical Reviews in Immunology*.

[5] Petroff M. G. (2005). Immune interactions at the maternal-fetal interface. *Journal of Reproductive Immunology*.

[6] Erlebacher A. (2013). Immunology of the maternal-fetal interface. *Annual Review of Immunology*.

[7] Gomez-Lopez N., StLouis D., Lehr M. A., Sanchez-Rodriguez E. N., Arenas-Hernandez M. (2014). Immune cells in term and preterm labor. *Cellular & Molecular Immunology*.

[8] PrabhuDas M., Bonney E., Caron K. (2015). Immune mechanisms at the maternal-fetal interface: perspectives and challenges. *Nature Immunology*.

[9] Bonney E. A. (2016). Immune regulation in pregnancy: a matter of perspective?. *Obstetrics and Gynecology Clinics of North America*.

[10] Gellersen B., Brosens I., Brosens J. (2007). Decidualization of the human endometrium: mechanisms, functions, and clinical perspectives. *Seminars in Reproductive Medicine*.

[11] Mori M., Bogdan A., Balassa T., Csabai T., Szekeres-Bartho J. (2016). The decidua-the maternal bed embracing the embryo-maintains the pregnancy. *Seminars in Immunopathology*.

[12] Gomez-Lopez N., Guilbert L. J., Olson D. M. (2010). Invasion of the leukocytes into the fetal-maternal interface during pregnancy. *Journal of Leukocyte Biology*.

[13] Moffett-King A. (2002). Natural killer cells and pregnancy. *Nature Reviews Immunology*.

[14] Croy B. A., Zhang J., Tayade C., Colucci F., Yadi H., Yamada A. T. (2010). Analysis of uterine natural killer cells in mice. *Methods in Molecular Biology*.

[15] Male V., Sharkey A., Masters L., Kennedy P. R., Farrell L. E., Moffett A. (2011). The effect of pregnancy on the uterine NK cell KIR repertoire. *European Journal of Immunology*.

[16] Gaynor L. M., Colucci F. (2017). Uterine natural killer cells: functional distinctions and influence on pregnancy in humans and mice. *Frontiers in Immunology*.

[17] Vento-Tormo R., Efremova M., Botting R. A. (2018). Single-cell reconstruction of the early maternal-fetal interface in humans. *Nature*.

[18] Hunt J. S., Manning L. S., Wood G. W. (1984). Macrophages in murine uterus are immunosuppressive. *Cellular Immunology*.

[19] Tawfik O. W., Hunt J. S., Wood G. W. (1986). Partial characterization of uterine cells responsible for suppression of murine maternal anti-fetal immune responses. *Journal of Reproductive Immunology*.

[20] Gustafsson C., Mjösberg J., Matussek A. (2008). Gene expression profiling of human decidual macrophages: evidence for immunosuppressive phenotype. *PLoS One*.

[21] Repnik U., Tilburgs T., Roelen D. L. (2008). Comparison of macrophage phenotype between decidua basalis and decidua parietalis by flow cytometry. *Placenta*.

[22] Svensson J., Jenmalm M. C., Matussek A., Geffers R., Berg G., Ernerudh J. (2011). Macrophages at the fetal-maternal interface express markers of alternative activation and are induced by M-CSF and IL-10. *The Journal of Immunology*.

[23] Houser B. L., Tilburgs T., Hill J., Nicotra M. L., Strominger J. L. (2011). Two unique human decidual macrophage populations. *The Journal of Immunology*.

[24] Kim S. Y., Romero R., Tarca A. L. (2012). Methylome of fetal and maternal monocytes and macrophages at the feto-maternal interface. *American Journal of Reproductive Immunology*.

[25] Hamilton S., Oomomian Y., Stephen G. (2012). Macrophages infiltrate the human and rat decidua during term and preterm labor: evidence that decidual inflammation precedes labor. *Biology of Reproduction*.

[26] Svensson-Arvelund J., Mehta R. B., Lindau R. (2015). The human fetal placenta promotes tolerance against the semiallogeneic fetus by inducing regulatory T cells and homeostatic M2 macrophages. *The Journal of Immunology*.

[27] Xu Y., Romero R., Miller D. (2016). An M1-like macrophage polarization in decidual tissue during spontaneous preterm labor that is attenuated by rosiglitazone treatment. *The Journal of Immunology*.

[28] Amsalem H., Kwan M., Hazan A. (2014). Identification of a novel neutrophil population: proangiogenic granulocytes in second-trimester human decidua. *The Journal of Immunology*.

[29] Nadkarni S., Smith J., Sferruzzi-Perri A. N. (2016). Neutrophils induce proangiogenic T cells with a regulatory phenotype in pregnancy. *Proceedings of the National Academy of Sciences of the United States of America*.

[30] Vacca P., Montaldo E., Croxatto D. (2015). Identification of diverse innate lymphoid cells in human decidua. *Mucosal Immunology*.

[31] Doisne J. M., Balmas E., Boulenouar S. (2015). Composition, development, and function of uterine innate lymphoid cells. *The Journal of Immunology*.

[32] Montaldo E., Vacca P., Chiossone L. (2016). Unique Eomes^+^ NK cell subsets are present in uterus and decidua during early pregnancy. *Frontiers in Immunology*.

[33] Croxatto D., Micheletti A., Montaldo E. (2016). Group 3 innate lymphoid cells regulate neutrophil migration and function in human decidua. *Mucosal Immunology*.

[34] Xu Y., Romero R., Miller D. (2018). Innate lymphoid cells at the human maternal-fetal interface in spontaneous preterm labor. *American Journal of Reproductive Immunology*.

[35] Miller D., Motomura K., Garcia-Flores V., Romero R., Gomez-Lopez N. (2018). Innate lymphoid cells in the maternal and fetal compartments. *Frontiers in Immunology*.

[36] Vargas M. L., Såntos J. L., Ruiz C. (1993). Comparison of the proportions of leukocytes in early and term human decidua. *American Journal of Reproductive Immunology*.

[37] Bonney E. A., Pudney J., Anderson D. J., Hill J. A. (2000). Gamma-delta T cells in midgestation human placental villi. *Gynecologic and Obstetric Investigation*.

[38] Sindram-Trujillo A. P., Scherjon S. A., Miert P. P. H. V., Kanhai H. H. H., Roelen D. L., Claas F. H. J. (2004). Comparison of decidual leukocytes following spontaneous vaginal delivery and elective cesarean section in uncomplicated human term pregnancy. *Journal of Reproductive Immunology*.

[39] Tilburgs T., Roelen D., Vandermast B. (2006). Differential distribution of CD4^+^CD25^bright^ and CD8^+^CD28^−^ T-cells in decidua and maternal blood during human pregnancy. *Placenta*.

[40] Constantin C. M., Masopust D., Gourley T. (2007). Normal establishment of virus-specific memory CD8 T cell pool following primary infection during pregnancy. *The Journal of Immunology*.

[41] Tilburgs T., Scherjon S. A., Roelen D. L., Claas F. H. J. (2009). Decidual CD8+CD28- T cells express CD103 but not perforin. *Human Immunology*.

[42] Tilburgs T., van der Mast B. J., Nagtzaam N. M. A., Roelen D. L., Scherjon S. A., Claas F. H. J. (2009). Expression of NK cell receptors on decidual T cells in human pregnancy. *Journal of Reproductive Immunology*.

[43] Norton M. T., Fortner K. A., Oppenheimer K. H., Bonney E. A. (2010). Evidence that CD8 T-cell homeostasis and function remain intact during murine pregnancy. *Immunology*.

[44] Tilburgs T., Schonkeren D., Eikmans M. (2010). Human decidual tissue contains differentiated CD8+ effector-memory T cells with unique properties. *The Journal of Immunology*.

[45] Gomez-Lopez N., Vadillo-Perez L., Hernandez-Carbajal A., Godines-Enriquez M., Olson D. M., Vadillo-Ortega F. (2011). Specific inflammatory microenvironments in the zones of the fetal membranes at term delivery. *American Journal of Obstetrics and Gynecology*.

[46] Gomez-Lopez N., Vega-Sanchez R., Castillo-Castrejon M., Romero R., Cubeiro-Arreola K., Vadillo-Ortega F. (2013). Evidence for a role for the adaptive immune response in human term parturition. *American Journal of Reproductive Immunology*.

[47] Shepard M. T., Bonney E. A. (2013). PD-1 regulates T cell proliferation in a tissue and subset-specific manner during normal mouse pregnancy. *Immunological Investigations*.

[48] Bonney E. A. (2017). Alternative theories: pregnancy and immune tolerance. *Journal of Reproductive Immunology*.

[49] Powell R. M., Lissauer D., Tamblyn J. (2017). Decidual T cells exhibit a highly differentiated phenotype and demonstrate potential fetal specificity and a strong transcriptional response to IFN. *The Journal of Immunology*.

[50] van der Zwan A., Bi K., Norwitz E. R. (2018). Mixed signature of activation and dysfunction allows human decidual CD8^+^ T cells to provide both tolerance and immunity. *Proceedings of the National Academy of Sciences of the United States of America*.

[51] Jensen F., Muzzio D., Soldati R., Fest S., Zenclussen A. C. (2013). Regulatory B10 cells restore pregnancy tolerance in a mouse model. *Biology of Reproduction*.

[52] Zenclussen A. C. (2013). Adaptive immune responses during pregnancy. *American Journal of Reproductive Immunology*.

[53] Fettke F., Schumacher A., Costa S. D., Zenclussen A. C. (2014). B cells: the old new players in reproductive immunology. *Frontiers in Immunology*.

[54] Leng Y., Romero R., Xu Y. (article e13102, 2019). Are B cells altered in the decidua of women with preterm or term labor?. *American Journal of Reproductive Immunology*.

[55] Romero R., Dey S. K., Fisher S. J. (2014). Preterm labor: one syndrome, many causes. *Science*.

[56] Arenas-Hernandez M., Romero R., St Louis D., Hassan S. S., Kaye E. B., Gomez-Lopez N. (2016). An imbalance between innate and adaptive immune cells at the maternal–fetal interface occurs prior to endotoxin-induced preterm birth. *Cellular & Molecular Immunology*.

[57] Blencowe H., Cousens S., Oestergaard M. Z. (2012). National, regional, and worldwide estimates of preterm birth rates in the year 2010 with time trends since 1990 for selected countries: a systematic analysis and implications. *Lancet*.

[58] Liu L., Oza S., Hogan D. (2015). Global, regional, and national causes of child mortality in 2000–13, with projections to inform post-2015 priorities: an updated systematic analysis. *The Lancet*.

[59] Manuck T. A., Rice M. M., Bailit J. L. (2016). Preterm neonatal morbidity and mortality by gestational age: a contemporary cohort. *American Journal of Obstetrics and Gynecology*.

[60] Gomez-Lopez N., Romero R., Arenas-Hernandez M. (2016). In vivo T-cell activation by a monoclonal *α*CD3*ε* antibody induces preterm labor and birth. *American Journal of Reproductive Immunology*.

[61] Arenas-Hernandez M., Romero R., Xu Y. (2019). Effector and activated T cells induce preterm labor and birth that is prevented by treatment with progesterone. *The Journal of Immunology*.

[62] Zajac A. J., Blattman J. N., Murali-Krishna K. (1998). Viral immune evasion due to persistence of activated T cells without effector function. *The Journal of Experimental Medicine*.

[63] Gallimore A., Glithero A., Godkin A. (1998). Induction and exhaustion of lymphocytic choriomeningitis virus-specific cytotoxic T lymphocytes visualized using soluble tetrameric major histocompatibility complex class I-peptide complexes. *The Journal of Experimental Medicine*.

[64] Barber D. L., Wherry E. J., Masopust D. (2006). Restoring function in exhausted CD8 T cells during chronic viral infection. *Nature*.

[65] Wherry E. J., Ha S. J., Kaech S. M. (2007). Molecular signature of CD8^+^ T cell exhaustion during chronic viral infection. *Immunity*.

[66] Blackburn S. D., Shin H., Haining W. N. (2009). Coregulation of CD8^+^ T cell exhaustion by multiple inhibitory receptors during chronic viral infection. *Nature Immunology*.

[67] Yi J. S., Cox M. A., Zajac A. J. (2010). T-cell exhaustion: characteristics, causes and conversion. *Immunology*.

[68] Wherry E. J. (2011). T cell exhaustion. *Nature Immunology*.

[69] Schietinger A., Greenberg P. D. (2014). Tolerance and exhaustion: defining mechanisms of T cell dysfunction. *Trends in Immunology*.

[70] Plunkett F. J., Franzese O., Finney H. M. (2007). The loss of telomerase activity in highly differentiated CD8^+^CD28^−^CD27^−^ T cells is associated with decreased Akt (Ser^473^) phosphorylation. *The Journal of Immunology*.

[71] Lanna A., Henson S. M., Escors D., Akbar A. N. (2014). The kinase p38 activated by the metabolic regulator AMPK and scaffold TAB1 drives the senescence of human T cells. *Nature Immunology*.

[72] Henson S. M., Lanna A., Riddell N. E. (2014). p38 signaling inhibits mTORC1-independent autophagy in senescent human CD8^+^ T cells. *The Journal of Clinical Investigation*.

[73] Wherry E. J., Kurachi M. (2015). Molecular and cellular insights into T cell exhaustion. *Nature Reviews Immunology*.

[74] Catakovic K., Klieser E., Neureiter D., Geisberger R. (2017). T cell exhaustion: from pathophysiological basics to tumor immunotherapy. *Cell Communication and Signaling*.

[75] Steger U., Denecke C., Sawitzki B., Karim M., Jones N. D., Wood K. J. (2008). Exhaustive differentiation of alloreactive CD8+ T cells: critical for determination of graft acceptance or rejection. *Transplantation*.

[76] Sarraj B., Ye J., Akl A. I. (2014). Impaired selectin-dependent leukocyte recruitment induces T-cell exhaustion and prevents chronic allograft vasculopathy and rejection. *Proceedings of the National Academy of Sciences of the United States of America*.

[77] Thorp E. B., Stehlik C., Ansari M. J. (2015). T-cell exhaustion in allograft rejection and tolerance. *Current Opinion in Organ Transplantation*.

[78] Akbar A. N., Henson S. M. (2011). Are senescence and exhaustion intertwined or unrelated processes that compromise immunity?. *Nature Reviews. Immunology*.

[79] Brenchley J. M., Karandikar N. J., Betts M. R. (2003). Expression of CD57 defines replicative senescence and antigen-induced apoptotic death of CD8^+^ T cells. *Blood*.

[80] Xu W., Larbi A. (2017). Markers of T cell senescence in humans. *International Journal of Molecular Sciences*.

[81] Romero R., Kim Y. M., Pacora P. (2018). The frequency and type of placental histologic lesions in term pregnancies with normal outcome. *Journal of Perinatal Medicine*.

[82] Redline R. W. (2006). Inflammatory responses in the placenta and umbilical cord. *Seminars in Fetal and Neonatal Medicine*.

[83] Kim C. J., Romero R., Chaemsaithong P., Chaiyasit N., Yoon B. H., Kim Y. M. (2015). Acute chorioamnionitis and funisitis: definition, pathologic features, and clinical significance. *American Journal of Obstetrics and Gynecology*.

[84] Redline R. W. (2015). Classification of placental lesions. *American Journal of Obstetrics and Gynecology*.

[85] Xu Y., Plazyo O., Romero R., Hassan S. S., Gomez-Lopez N. (2015). Isolation of leukocytes from the human maternal-fetal Interface. *Journal of Visualized Experiments*.

[86] Benjamini Y., Heller R. (2008). Screening for partial conjunction hypotheses. *Biometrics*.

[87] Cleveland W. S., Grosse E., Shyu W. M., Chambers J. M., Hastie T. J. (1992). Local regression models. *Statistical Models*.

[88] Team, RC (2016). *R: A Language and Environment for Statistical Computing*.

[89] Gomez-Lopez N., Laresgoiti-Servitje E. (2012). T regulatory cells: regulating both term and preterm labor?. *Immunology and Cell Biology*.

[90] Gomez-Lopez N., Olson D. M., Robertson S. A. (2016). Interleukin-6 controls uterine Th9 cells and CD8^+^ T regulatory cells to accelerate parturition in mice. *Immunology and Cell Biology*.

[91] Gomez-Lopez N., Romero R., Arenas-Hernandez M. (2017). *In vivo* activation of invariant natural killer T cells induces systemic and local alterations in T-cell subsets prior to preterm birth. *Clinical & Experimental Immunology*.

[92] Frascoli M., Coniglio L., Witt R. (2018). Alloreactive fetal T cells promote uterine contractility in preterm labor via IFN-*γ* and TNF-*α*. *Science Translational Medicine*.

[93] Boldison J., Chu C. J., Copland D. A. (2014). Tissue-resident exhausted effector memory CD8^+^ T cells accumulate in the retina during chronic experimental autoimmune uveoretinitis. *The Journal of Immunology*.

[94] Reiser J., Banerjee A. (2016). Effector, memory, and dysfunctional CD8^+^ T cell fates in the antitumor immune response. *Journal of Immunology Research*.

[95] Wang S. C., Li Y. H., Piao H. L. (2015). PD-1 and Tim-3 pathways are associated with regulatory CD8^+^ T-cell function in decidua and maintenance of normal pregnancy. *Cell Death & Disease*.

[96] Wang S., Zhu X. Y., Xu Y. Y. (2016). Programmed cell death-1 (PD-1) and T-cell immunoglobulin mucin-3 (Tim-3) regulate CD4+ T cells to induce type 2 helper T cell (Th2) bias at the maternal-fetal interface. *Human Reproduction*.

[97] Solders M., Gorchs L., Gidlöf S., Tiblad E., Lundell A. C., Kaipe H. (2017). Maternal adaptive immune cells in decidua parietalis display a more activated and coinhibitory phenotype compared to decidua basalis. *Stem Cells International*.

[98] Francisco L. M., Sage P. T., Sharpe A. H. (2010). The PD-1 pathway in tolerance and autoimmunity. *Immunological Reviews*.

[99] Baas M., Besançon A., Goncalves T. (2016). TGF*β*-dependent expression of PD-1 and PD-L1 controls CD8+ T cell anergy in transplant tolerance. *eLife*.

[100] Anderson A. C., Joller N., Kuchroo V. K. (2016). Lag-3, Tim-3, and TIGIT: co-inhibitory receptors with specialized functions in immune regulation. *Immunity*.

[101] Nagamatsu T., Schust D. J., Sugimoto J., Barrier B. F. (2009). Human decidual stromal cells suppress cytokine secretion by allogenic CD4^+^ T cells via PD-1 ligand interactions. *Human Reproduction*.

[102] Sayama S., Nagamatsu T., Schust D. J. (2013). Human decidual macrophages suppress IFN-*γ* production by T cells through costimulatory B7-H1:PD-1 signaling in early pregnancy. *Journal of Reproductive Immunology*.

[103] Hu X. H., Tang M. X., Mor G., Liao A. H. (2016). Tim-3: expression on immune cells and roles at the maternal-fetal interface. *Journal of Reproductive Immunology*.

[104] Xu Y. Y., Wang S. C., Lin Y. K., Li D. J., Du M. R. (2017). Tim-3 and PD-1 regulate CD8^+^ T cell function to maintain early pregnancy in mice. *Journal of Reproduction and Development*.

[105] Zhuang X., Xia X., Liu L., Zhang Y., Zhang X., Wang C. (2018). Expression of Tim-3 in peripheral blood mononuclear cells and placental tissue in unexplained recurrent spontaneous abortion. *Medicine*.

[106] Keelan J. A., Marvin K. W., Sato T. A., Coleman M., McCowan L. M. E., Mitchell M. D. (1999). Cytokine abundance in placental tissues: evidence of inflammatory activation in gestational membranes with term and preterm parturition. *American Journal of Obstetrics and Gynecology*.

[107] Young A., Thomson A. J., Ledingham M. A., Jordan F., Greer I. A., Norman J. E. (2002). Immunolocalization of proinflammatory cytokines in myometrium, cervix, and fetal membranes during human parturition at term. *Biology of Reproduction*.

[108] Osman I., Young A., Ledingham M. A. (2003). Leukocyte density and pro-inflammatory cytokine expression in human fetal membranes, decidua, cervix and myometrium before and during labour at term. *Molecular Human Reproduction*.

[109] Haddad R., Tromp G., Kuivaniemi H. (2006). Human spontaneous labor without histologic chorioamnionitis is characterized by an acute inflammation gene expression signature. *American Journal of Obstetrics and Gynecology*.

[110] Gomez-Lopez N., Estrada-Gutierrez G., Jimenez-Zamudio L., Vega-Sanchez R., Vadillo-Ortega F. (2009). Fetal membranes exhibit selective leukocyte chemotaxic activity during human labor. *Journal of Reproductive Immunology*.

[111] Nhan-Chang C. L., Romero R., Tarca A. L. (2010). Characterization of the transcriptome of chorioamniotic membranes at the site of rupture in spontaneous labor at term. *American Journal of Obstetrics and Gynecology*.

[112] Hamilton S. A., Tower C. L., Jones R. L. (2013). Identification of chemokines associated with the recruitment of decidual leukocytes in human labour: potential novel targets for preterm labour. *PLoS One*.

[113] Bukowski R., Sadovsky Y., Goodarzi H. (2017). Onset of human preterm and term birth is related to unique inflammatory transcriptome profiles at the maternal fetal interface. *PeerJ*.

[114] Saeidi A., Zandi K., Cheok Y. Y. (2018). T-cell exhaustion in chronic infections: reversing the state of exhaustion and reinvigorating optimal protective immune responses. *Frontiers in Immunology*.

[115] Gross G., Imamura T., Vogt S. K. (2000). Inhibition of cyclooxygenase-2 prevents inflammation-mediated preterm labor in the mouse. *American Journal of Physiology-Regulatory, Integrative and Comparative Physiology*.

[116] Hirsch E., Filipovich Y., Mahendroo M. (2006). Signaling via the type I IL-1 and TNF receptors is necessary for bacterially induced preterm labor in a murine model. *American Journal of Obstetrics and Gynecology*.

[117] Gonzalez J. M., Xu H., Chai J., Ofori E., Elovitz M. A. (2009). Preterm and term cervical ripening in CD1 mice (Mus musculus): similar or divergent molecular mechanisms?. *Biology of Reproduction*.

[118] Holt R., Timmons B. C., Akgul Y., Akins M. L., Mahendroo M. (2011). The molecular mechanisms of cervical ripening differ between term and preterm birth. *Endocrinology*.

[119] Willcockson A. R., Nandu T., Liu C. L., Nallasamy S., Kraus W. L., Mahendroo M. (2018). Transcriptome signature identifies distinct cervical pathways induced in lipopolysaccharide-mediated preterm birth. *Biology of Reproduction*.

[120] Ferguson M. G., Rhodes P. G., Morrison J. C., Puckett C. M. (1985). Clinical amniotic fluid infection and its effect on the neonate. *American Journal of Obstetrics and Gynecology*.

[121] Romero R., Mazor M., Wu Y. K. (1988). Infection in the pathogenesis of preterm labor. *Seminars in Perinatology*.

[122] Gomez R., Romero R., Edwin S. S., David C. (1997). Pathogenesis of preterm labor and preterm premature rupture of membranes associated with intraamniotic infection. *Infectious Disease Clinics of North America*.

[123] Romero R., Gomez R., Chaiworapongsa T., Conoscenti G., Cheol Kim J., Mee Kim Y. (2001). The role of infection in preterm labour and delivery. *Paediatric and Perinatal Epidemiology*.

[124] Yoon B. H., Romero R., Moon J. B. (2001). Clinical significance of intra-amniotic inflammation in patients with preterm labor and intact membranes. *American Journal of Obstetrics and Gynecology*.

[125] Romero R., Gotsch F., Pineles B., Kusanovic J. P. (2007). Inflammation in pregnancy: its roles in reproductive physiology, obstetrical complications, and fetal injury. *Nutrition Reviews*.

[126] Kemp M. W. (2014). Preterm birth, intrauterine infection, and fetal inflammation. *Frontiers in Immunology*.

[127] Keelan J. A. (2018). Intrauterine inflammatory activation, functional progesterone withdrawal, and the timing of term and preterm birth. *Journal of Reproductive Immunology*.

[128] Romero R., Miranda J., Chaiworapongsa T. (2014). Prevalence and clinical significance of sterile intra-amniotic inflammation in patients with preterm labor and intact membranes. *American Journal of Reproductive Immunology*.

[129] Romero R., Miranda J., Chaemsaithong P. (2015). Sterile and microbial-associated intra-amniotic inflammation in preterm prelabor rupture of membranes. *The Journal of Maternal-Fetal & Neonatal Medicine*.

[130] Oh K. J., Kim S. M., Hong J. S. (2017). Twenty-four percent of patients with clinical chorioamnionitis in preterm gestations have no evidence of either culture-proven intraamniotic infection or intraamniotic inflammation. *American Journal of Obstetrics and Gynecology*.

[131] Gomez-Lopez N., Romero R., Panaitescu B. (2018). Inflammasome activation during spontaneous preterm labor with intra-amniotic infection or sterile intra-amniotic inflammation. *American Journal of Reproductive Immunology*.

[132] Blattman J. N., Grayson J. M., Wherry E. J., Kaech S. M., Smith K. A., Ahmed R. (2003). Therapeutic use of IL-2 to enhance antiviral T-cell responses in vivo. *Nature Medicine*.

[133] Ejrnaes M., Filippi C. M., Martinic M. M. (2006). Resolution of a chronic viral infection after interleukin-10 receptor blockade. *The Journal of Experimental Medicine*.

[134] Blackburn S. D., Wherry E. J. (2007). IL-10, T cell exhaustion and viral persistence. *Trends in Microbiology*.

[135] Ni G., Wang T., Walton S. (2015). Manipulating IL-10 signalling blockade for better immunotherapy. *Cellular Immunology*.

[136] Freeman G. J., Wherry E. J., Ahmed R., Sharpe A. H. (2006). Reinvigorating exhausted HIV-specific T cells via PD-1-PD-1 ligand blockade. *The Journal of Experimental Medicine*.

[137] Penaloza-MacMaster P., Kamphorst A. O., Wieland A. (2014). Interplay between regulatory T cells and PD-1 in modulating T cell exhaustion and viral control during chronic LCMV infection. *The Journal of Experimental Medicine*.

[138] Topalian S. L., Drake C. G., Pardoll D. M. (2015). Immune checkpoint blockade: a common denominator approach to cancer therapy. *Cancer Cell*.

[139] Jiang Y., Li Y., Zhu B. (2015). T-cell exhaustion in the tumor microenvironment. *Cell Death & Disease*.

[140] Lee J., Ahn E., Kissick H. T., Ahmed R. (2015). Reinvigorating exhausted T cells by blockade of the PD-1 pathway. *Forum on Immunopathological Diseases and Therapeutics*.

[141] Zarour H. M. (2016). Reversing T-cell dysfunction and exhaustion in cancer. *Clinical Cancer Research*.

[142] Chou J. P., Effros R. B. (2013). T cell replicative senescence in human aging. *Current Pharmaceutical Design*.

[143] Akbar A. N., Henson S. M., Lanna A. (2016). Senescence of T lymphocytes: implications for enhancing human immunity. *Trends in Immunology*.

[144] Hirota Y., Daikoku T., Tranguch S., Xie H., Bradshaw H. B., Dey S. K. (2010). Uterine-specific p53 deficiency confers premature uterine senescence and promotes preterm birth in mice. *Journal of Clinical Investigation*.

[145] Gomez-Lopez N., Romero R., Plazyo O. (2017). Preterm labor in the absence of acute histologic chorioamnionitis is characterized by cellular senescence of the chorioamniotic membranes. *American Journal of Obstetrics and Gynecology*.

[146] Hirota Y., Cha J., Yoshie M., Daikoku T., Dey S. K. (2011). Heightened uterine mammalian target of rapamycin complex 1 (mTORC1) signaling provokes preterm birth in mice. *Proceedings of the National Academy of Sciences of the United States of America*.

[147] Phillippe M. (2015). Cell-free fetal DNA, telomeres, and the spontaneous onset of parturition. *Reproductive Sciences*.

[148] Deng W., Cha J., Yuan J. (2016). p53 coordinates decidual sestrin 2/AMPK/mTORC1 signaling to govern parturition timing. *The Journal of Clinical Investigation*.

[149] Bonney E. A. (2017). Mapping out p38MAPK. *American Journal of Reproductive Immunology*.

[150] Richardson L., Dixon C. L., Aguilera-Aguirre L., Menon R. (2018). Oxidative stress-induced TGF-beta/TAB1-mediated p38MAPK activation in human amnion epithelial cells. *Biology of Reproduction*.

[151] Warrington K. J., Vallejo A. N., Weyand C. M., Goronzy J. J. (2003). CD28 loss in senescent CD4^+^ T cells: reversal by interleukin-12 stimulation. *Blood*.

[152] Fauce S. R., Jamieson B. D., Chin A. C. (2008). Telomerase-based pharmacologic enhancement of antiviral function of human CD8^+^ T lymphocytes. *The Journal of Immunology*.

[153] Di Mitri D., Azevedo R. I., Henson S. M. (2011). Reversible senescence in human CD4^+^CD45RA^+^CD27^–^ memory T cells. *The Journal of Immunology*.

