# Supplementary material for: Exhausted and Senescent T Cells at the Maternal-Fetal Interface in Preterm and Term Labor
Source: J Immunol Res. 2019 May 23;2019:3128010. doi: 10.1155/2019/3128010 (PMC6556261; doi:10.1155/2019/3128010)
Supplement: Supplementary Materials — Supplementary Table 1: antibodies used for immunophenotyping and cell sorting. Supplementary Table 2: literature review performed to select markers for identifying exhausted and senescent T cells. Supplementary Figure 1: exhausted and senescent CD4+ and CD8+ T cells display an effector memory phenotype in the decidua basalis and decidua parietalis. The proportions of exhausted and senescent CD4+ and CD8+ T cells within the effector memory (TEM) (A-H) or terminally differentiated effector memory (TEMRA) (I-L) T cell subsets in the decidua basalis and decidua parietalis from women who delivered preterm with labor (PTL) or without labor (PTNL) and women who delivered at term with labor (TIL) or without labor (TNL). N = 8–21 per group. Red midlines and whiskers indicate medians and interquartile ranges, respectively. [file 3128010.f1.pdf]

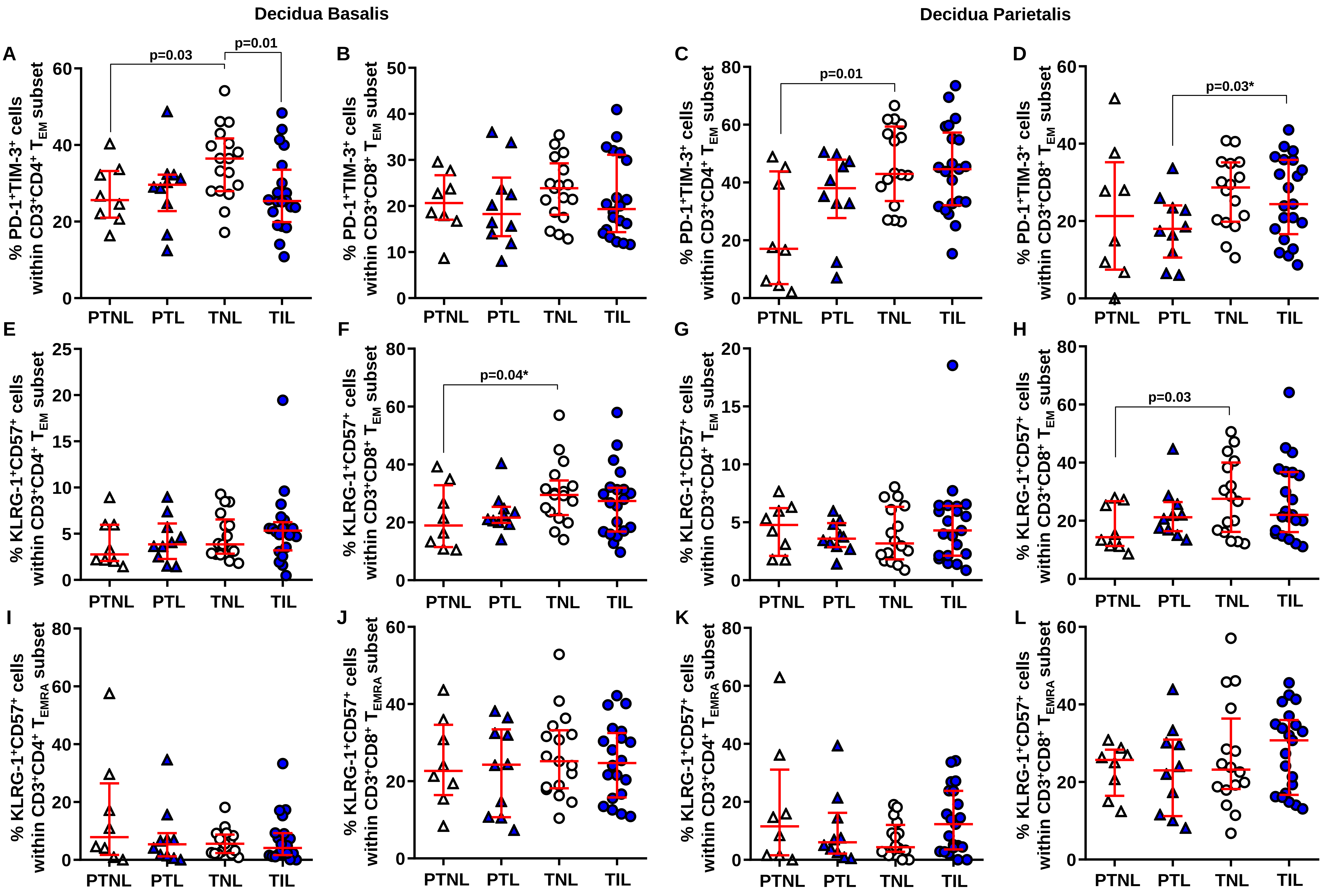

Supplementary Figure 1. Exhausted and senescent CD4+ and CD8+ T cells display an effector memory phenotype in the decidua basalis and decidua parietalis. The proportions of exhausted and senescent CD4+ and CD8+ T cells within the effector memory (T_{EM}) (**A-H**) or terminally differentiated effector memory (T_{EMRA}) (**I-L**) T-cell subsets in the decidua basalis and parietalis from women who delivered preterm with labor (PTL) or without labor (PTNL) and women who delivered at term with labor (TIL) or without labor (TNL). N = 8 – 21 per group. Red midlines and whiskers indicate medians and interquartile ranges, respectively.

Supplementary Table 1. Antibodies used for immunophenotyping and cell sorting

Antigen	Fluorophore	Clone	Company
CD45	APC-H7	2D1	BD Biosciences
CD8	BUV395 / PE	RPA-T8 / H1T8a	BD Biosciences
CD4	BUV737 / PerCP-Cy5.5	SK3 / RPA-T4	BD Biosciences
CD3	PerCP-Cy5.5 / BV421	SK7 / UCHT1	BD Biosciences
CD56	BV711	NCAM16.2	BD Biosciences
PD-1	PE-CF594	EH12.1	BD Biosciences
KLRG-1	PE	13A2	Invitrogen
CD57	APC-H7 / APC	NK-1	BD Biosciences
LAG-3	BV421	T47-530	BD Biosciences
TIM-3	BB515	7D3	BD Biosciences
CTLA-4	PE-Cy5	BNI3	BD Biosciences
IFNγ	BV650	4S.B3	BD Biosciences
TNFα	BV605	MAb11	BD Biosciences
CD45RA	AlexaFluor700	HI100	BD Biosciences
CCR7	PE-Cy7	3D12	BD Biosciences

Supplementary Table 2. Literature review performed to select markers for identifying exhausted and senescent T cells

T cells subtype	Markers	References
**Exhausted T cells**	**PD-1**	Day C et.al [1], Barber D et.al [2], Okazaki T et.al [3], Wherry E et.al [4], Blackburn S et.al [5], Yi J et.al [6], Jin H et.al [7], Rangachari M et.al [8], Ferris R et.al [9], Pauken K et.al [10], Turnis M et.al [11], Ozkazanc D et.al. [12], Li J et.al [13], Shayan G et.al [14], Li Z et.al [15], Taghiloo S et.al [16], Ma Q et.al [17], Wei S et.al [18], Grabmeier-Pfistershammer K et.al [19], Tan J et.al [20, 21], Nakano M et.al [22], Liu L et.al [23], Liu Z et.al [24], Wang X et.al [25]
	**TIM-3**	Yi J et.al [6], Jin H et.al [7], Ngjow S et.al [26], Rangachari M et.al [8], Ferris R et.al [9], Ozkazanc D et.al. [12], Li J et.al [13], Jayaraman P et.al [27], Shayan G et.al [14], Li Z et.al [15], Taghiloo S et.al [16], Ma Q et.al [17], Tan J et.al [21], Nakano M et.al [22], Liu L et.al [23], Liu Z et.al [24], He Y et.al [28], Wang X et.al [25].
	**CTLA-4**	Day C et.al [1], Wherry E et.al [4], Blackburn S et.al [5], Yi J et.al [6], Herrmann A et.al [29], Turnis M et.al [11], Ozkazanc D et.al [12], Wei S et.al [18], Grabmeier-Pfistershammer K et.al [19].
	**LAG-3**	Richter K et.al [30], Wherry E et.al [4], Yi J et.al [6], Blackburn S et.al [5], Ma Q et.al [17], Turnis M et.al [11], Ferris R et.al [9], Ozkazanc D et.al. [12]
**Senescent T cells**	**CD57**	Brenchley J et.al [31], Simpson R et.al [32], Tae H et.al [33], Kared H et.al [34], Suen H et.al [35], Tan J et.al [20], Cura P et.al [36], Lee Y et.al [37], Duggal N et.al [38], Heath J et.al [39].
	**KLRG-1**	Voehringer D et.al [40], Ouyang Q et.al [41], Heffner M et.al [42], Simpson R et.al [32], Henson S et.al [43], Prlic M et.al [44], Göthert J et.al [45], Pauken K et.al [10], Suen H et.al [35], Cura P et.al [36].

References

1. Day, C.L., et al., *PD-1 expression on HIV-specific T cells is associated with T-cell exhaustion and disease progression.* Nature, 2006. **443**(7109): p. 350-4.
2. Barber, D.L., et al., *Restoring function in exhausted CD8 T cells during chronic viral infection.* Nature, 2006. **439**(7077): p. 682-7.
3. Okazaki, T. and T. Honjo, *Rejuvenating exhausted T cells during chronic viral infection.* Cell, 2006. **124**(3): p. 459-61.
4. Wherry, E.J., et al., *Molecular signature of CD8+ T cell exhaustion during chronic viral infection.* Immunity, 2007. **27**(4): p. 670-84.
5. Blackburn, S.D., et al., *Coregulation of CD8+ T cell exhaustion by multiple inhibitory receptors during chronic viral infection.* Nat Immunol, 2009. **10**(1): p. 29-37.

6. Yi, J.S., M.A. Cox, and A.J. Zajac, *T-cell exhaustion: characteristics, causes and conversion.* Immunology, 2010. **129**(4): p. 474-81.
7. Jin, H.T., et al., *Cooperation of Tim-3 and PD-1 in CD8 T-cell exhaustion during chronic viral infection.* Proc Natl Acad Sci U S A, 2010. **107**(33): p. 14733-8.
8. Rangachari, M., et al., *Bat3 promotes T cell responses and autoimmunity by repressing Tim-3-mediated cell death and exhaustion.* Nat Med, 2012. **18**(9): p. 1394-400.
9. Ferris, R.L., B. Lu, and L.P. Kane, *Too much of a good thing? Tim-3 and TCR signaling in T cell exhaustion.* J Immunol, 2014. **193**(4): p. 1525-30.
10. Pauken, K.E. and E.J. Wherry, *Overcoming T cell exhaustion in infection and cancer.* Trends Immunol, 2015. **36**(4): p. 265-76.
11. Turnis, M.E., L.P. Andrews, and D.A. Vignali, *Inhibitory receptors as targets for cancer immunotherapy.* Eur J Immunol, 2015. **45**(7): p. 1892-905.
12. Ozkazanc, D., et al., *Functional exhaustion of CD4(+) T cells induced by co-stimulatory signals from myeloid leukaemia cells.* Immunology, 2016. **149**(4): p. 460-471.
13. Li, J., et al., *Tumor-infiltrating Tim-3(+) T cells proliferate avidly except when PD-1 is co-expressed: Evidence for intracellular cross talk.* Oncoimmunology, 2016. **5**(10): p. e1200778.
14. Shayan, G., et al., *Adaptive resistance to anti-PD1 therapy by Tim-3 upregulation is mediated by the PI3K-Akt pathway in head and neck cancer.* Oncoimmunology, 2017. **6**(1): p. e1261779.
15. Li, Z., et al., *TIM-3 plays a more important role than PD-1 in the functional impairments of cytotoxic T cells of malignant Schwannomas.* Tumour Biol, 2017. **39**(5): p. 1010428317698352.
16. Taghiloo, S., et al., *Frequency and functional characterization of exhausted CD8(+) T cells in chronic lymphocytic leukemia.* Eur J Haematol, 2017. **98**(6): p. 622-631.
17. Ma, Q.Y., et al., *Function and regulation of LAG3 on CD4(+)CD25(-) T cells in non-small cell lung cancer.* Exp Cell Res, 2017. **360**(2): p. 358-364.
18. Wei, S.C., et al., *Distinct Cellular Mechanisms Underlie Anti-CTLA-4 and Anti-PD-1 Checkpoint Blockade.* Cell, 2017. **170**(6): p. 1120-1133 e17.
19. Grabmeier-Pfistershammer, K., et al., *Antibodies targeting BTLA or TIM-3 enhance HIV-1 specific T cell responses in combination with PD-1 blockade.* Clin Immunol, 2017. **183**: p. 167-173.
20. Tan, J., et al., *Higher PD-1 expression concurrent with exhausted CD8+ T cells in patients with de novo acute myeloid leukemia.* Chin J Cancer Res, 2017. **29**(5): p. 463-470.
21. Tan, J., et al., *Increased exhausted CD8(+) T cells with programmed death-1, T-cell immunoglobulin and mucin-domain-containing-3 phenotype in patients with multiple myeloma.* Asia Pac J Clin Oncol, 2018.
22. Nakano, M., et al., *PD-1+ TIM-3+ T cells in malignant ascites predict prognosis of gastrointestinal cancer.* Cancer Sci, 2018. **109**(9): p. 2986-2992.
23. Liu, L., et al., *T cell exhaustion characterized by compromised MHC class I and II restricted cytotoxic activity associates with acute B lymphoblastic leukemia*

relapse after allogeneic hematopoietic stem cell transplantation. Clin Immunol, 2018. **190**: p. 32-40.
24. Liu, Z., et al., *Novel Effector Phenotype of Tim-3(+) Regulatory T Cells Leads to Enhanced Suppressive Function in Head and Neck Cancer Patients.* Clin Cancer Res, 2018. **24**(18): p. 4529-4538.
25. Wang, X., et al., *Exosomes derived from exhausted CD8+ T cells impaired the anticancer function of normal CD8+ T cells.* J Med Genet, 2019. **56**(1): p. 29-31.
26. Ngiow, S.F., et al., *Anti-TIM3 antibody promotes T cell IFN-gamma-mediated antitumor immunity and suppresses established tumors.* Cancer Res, 2011. **71**(10): p. 3540-51.
27. Jayaraman, P., et al., *TIM3 Mediates T Cell Exhaustion during Mycobacterium tuberculosis Infection.* PLoS Pathog, 2016. **12**(3): p. e1005490.
28. He, Y., et al., *TIM-3, a promising target for cancer immunotherapy.* Onco Targets Ther, 2018. **11**: p. 7005-7009.
29. Herrmann, A., et al., *CTLA4 aptamer delivers STAT3 siRNA to tumor-associated and malignant T cells.* J Clin Invest, 2014. **124**(7): p. 2977-87.
30. Richter, K., P. Agnellini, and A. Oxenius, *On the role of the inhibitory receptor LAG-3 in acute and chronic LCMV infection.* Int Immunol, 2010. **22**(1): p. 13-23.
31. Brenchley, J.M., et al., *Expression of CD57 defines replicative senescence and antigen-induced apoptotic death of CD8+ T cells.* Blood, 2003. **101**(7): p. 2711-20.
32. Simpson, R.J., et al., *Senescent T-lymphocytes are mobilised into the peripheral blood compartment in young and older humans after exhaustive exercise.* Brain Behav Immun, 2008. **22**(4): p. 544-51.
33. Tae Yu, H., et al., *Characterization of CD8(+)CD57(+) T cells in patients with acute myocardial infarction.* Cell Mol Immunol, 2015. **12**(4): p. 466-73.
34. Kared, H., et al., *CD57 in human natural killer cells and T-lymphocytes.* Cancer Immunol Immunother, 2016. **65**(4): p. 441-52.
35. Suen, H., et al., *Multiple myeloma causes clonal T-cell immunosenescence: identification of potential novel targets for promoting tumour immunity and implications for checkpoint blockade.* Leukemia, 2016. **30**(8): p. 1716-24.
36. Cura Daball, P., et al., *CD57 identifies T cells with functional senescence before terminal differentiation and relative telomere shortening in patients with activated PI3 kinase delta syndrome.* Immunol Cell Biol, 2018. **96**(10): p. 1060-1071.
37. Lee, Y.H., et al., *Senescent T Cells Predict the Development of Hyperglycemia in Humans.* Diabetes, 2018.
38. Duggal, N.A., et al., *Major features of immunesenescence, including reduced thymic output, are ameliorated by high levels of physical activity in adulthood.* Aging Cell, 2018. **17**(2).
39. Heath, J.J., et al., *Proximity of Cytomegalovirus-Specific CD8(+) T Cells to Replicative Senescence in Human Immunodeficiency Virus-Infected Individuals.* Front Immunol, 2018. **9**: p. 201.
40. Voehringer, D., M. Koschella, and H. Pircher, *Lack of proliferative capacity of human effector and memory T cells expressing killer cell lectinlike receptor G1 (KLRG1).* Blood, 2002. **100**(10): p. 3698-702.

41. Ouyang, Q., et al., *Age-associated accumulation of CMV-specific CD8+ T cells expressing the inhibitory killer cell lectin-like receptor G1 (KLRG1).* Exp Gerontol, 2003. **38**(8): p. 911-20.
42. Heffner, M. and D.T. Fearon, *Loss of T cell receptor-induced Bmi-1 in the KLRG1(+) senescent CD8(+) T lymphocyte.* Proc Natl Acad Sci U S A, 2007. **104**(33): p. 13414-9.
43. Henson, S.M., et al., *KLRG1 signaling induces defective Akt (ser473) phosphorylation and proliferative dysfunction of highly differentiated CD8+ T cells.* Blood, 2009. **113**(26): p. 6619-28.
44. Prlic, M., J.A. Sacks, and M.J. Bevan, *Dissociating markers of senescence and protective ability in memory T cells.* PLoS One, 2012. **7**(3): p. e32576.
45. Gothert, J.R., et al., *Expanded CD8+ T cells of murine and human CLL are driven into a senescent KLRG1+ effector memory phenotype.* Cancer Immunol Immunother, 2013. **62**(11): p. 1697-1709.
